# Supplementary material for: Skyrmion pinning energetics in thin film systems
Source: Nat Commun. 2022 Jun 6;13:3144. doi: 10.1038/s41467-022-30743-4 (PMC9170736; doi:10.1038/s41467-022-30743-4)
Supplement: Supplementary file 1 — Supplementary Information [file 41467_2022_30743_MOESM1_ESM.pdf]

Skyrmion pinning energetics in thin film systems

Supplementary Information

Raphael Gruber[1], Jakub Zázvorka[2], Maarten A. Brems[1], Davi R. Rodrigues[1,3,4], Takaaki Dohi[1], Nico Kerber[1], Boris Seng[1,5], Mehran Vafaee[1,6], Karin Everschor-Sitte[1,4,7], Peter Virnau[1], Mathias Kläui*[1]

[1] Institute of Physics, Johannes Gutenberg-Universität Mainz, Staudingerweg 7, Mainz 55128, Germany
[2] Institute of Physics, Faculty of Mathematics and Physics, Charles University, Ke Karlovu 5, Prague 12116, Czech Republic
[3] Dipartimento di Ingegneria Elettrica e dell'Informazione, Politecnico di Bari,Via E. Orabona 4, Bari 70125, Italy
[4] Faculty of Physics, University of Duisburg-Essen, Lotharstraße 1, Duisburg 47057, Germany
[5] Institut Jean Lamour, UMR CNRS 7198, Université de Lorraine, 2 allée André Guinier, Nancy 54011, France
[6] Singulus Technologies AG, Hanauer Landstraße 103, Kahl am Main 63796, Germany
[7] Center for Nanointegration Duisburg- Essen (CENIDE), University of Duisburg-Essen, Carl-Benz-Straße 199, Duisburg 47057, Germany
*E-mail: klaeui@uni-mainz.de

Supplementary Notes

Supplementary Note 1

Supplementary Fig. 1 provides additional information regarding the skyrmion occurrences in the thin film sample. Besides the probability of finding a skyrmion at certain positions, it shows the positions of skyrmions in the beginning and at the end of the observations in Supplementary Fig. 1a. Whereas the newly nucleated skyrmions (blue dots) are evenly spread over the sample, the skyrmions at the end of every video (red dots) are as expected often found at pinning sites. The histograms along the x and y axis indicate furthermore that the skyrmion distribution is uniform along the sample axes throughout the measurement time.

Within the observed area, the positions featuring strong pinning appear evenly spread. The green annotations in Supplementary Fig. 1a exemplarily provide a few distances in micrometers between pinning spots. To study the spatial distribution of pinning sites, we therefore also look at the Fourier transformation of the histogram of skyrmion center positions. The radial dependence of the FFT intensity is depicted in Supplementary Fig. 1b as a function of real space distances. The distances denoted in Supplementary Fig. 1a are indicated by the green solid lines and lay within the FFT region with high intensities. This indicates a range of characteristic distances between positions at which skyrmions appear for a significant amount of time. We therefore conclude the existence of typical lengths under which strong pinning centers occur. In the analysis, we restrict ourselves to the radial dependence of the FFT since the angular distribution is homogeneous meaning that there is no preferred axis in the sample along which pinning sites are observed.

The detailed investigation of the underlying physical reason for this repeated appearance of pinning centers is beyond the scope of this paper. However, since locally varying material parameters result in the occurrence of pinning sites, this effect is potentially featured by a periodic appearance of impurities which can be governed by the physics of the manufacturing procedure of the amorphous multilayer stack. Therefore, the sample growth must be considered when fabricating a stack with specific pinning properties.

Supplementary Note 2

Skyrmions at pinning site 1 can appear at slightly varying positions depending on the skyrmion size. Supplementary Fig. 2a shows skyrmion boundaries for two different occurring skyrmion center of mass positions, which are indicated in Supplementary Fig. 2b. The SB position coincides at one side for both skyrmion sizes indicating a pinned behavior. However, the extension is different on the other side and determined by the size. Therefore, different skyrmion center positions can occur at this particular site (see Supplementary Fig. 2b). The size difference belonging to this variation of the center positions is visible in Supplementary Fig. 2c.

Supplementary Note 3

The small skyrmions appear between the regions of lowered anisotropy introduced in the simulation. Supplementary Fig. 3a shows the probability map of skyrmion center occurrences as presented in Fig. 5. Supplementary Fig. 3c,e show the perpendicular magnetization component of two such simulated skyrmions at different positions exemplarily. As proposed by our SB pinning concept, the area with reduced anisotropy is thereby occupied mainly by the SB depicted in white. Similarly, Supplementary Fig. 3b,d,f show the center occurrence probability and magnetization components along the z-direction for two simulated skyrmions, respectively. Now, the large skyrmions do not fit between the boxes anymore with their core. Instead, they arrange above or below to maximize the overlap of their SB with the reduced anisotropy region while keeping the same overlap small for their core.

During that process, the skyrmions are also deformed with average eccentricities of 0.05 and 0.11 for the small and the large skyrmion, respectively.

Supplementary Figures

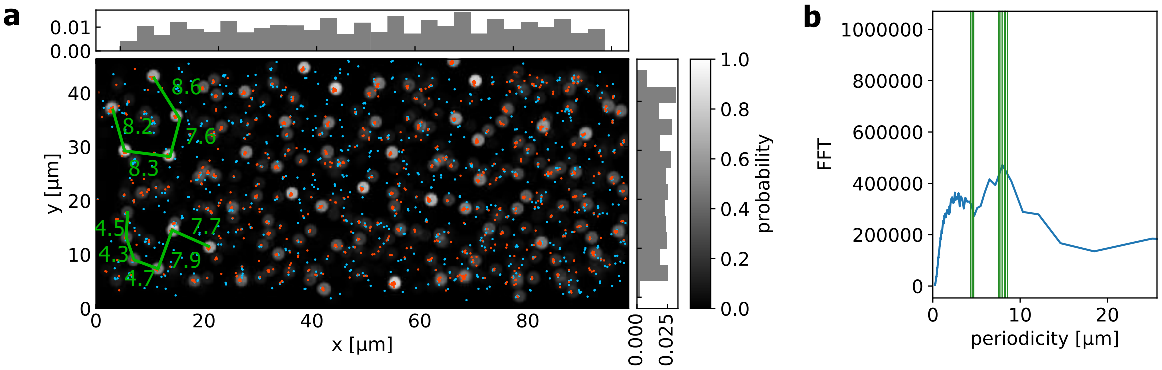

Supplementary Fig. 1. Occurrence of the pinning. *(a) The greyscale image shows the probability of skyrmions covering certain pixels as established in Fig. 1. The blue and red scattered spots depict the skyrmion center positions at the beginning and end of every video, respectively. The normalized histograms on the top and right show the uniformity of the of x- and y- coordinate distribution of every observed skyrmion center. The green lines with annotations in micrometers show distances between arbitrarily selected pinning sites. In (b), the radial part of the FFT intensities of the skyrmion occurrences from (a) is depicted whereby the frequencies are rescaled to real space distances. The green lines indicate the distances denoted in (a).*

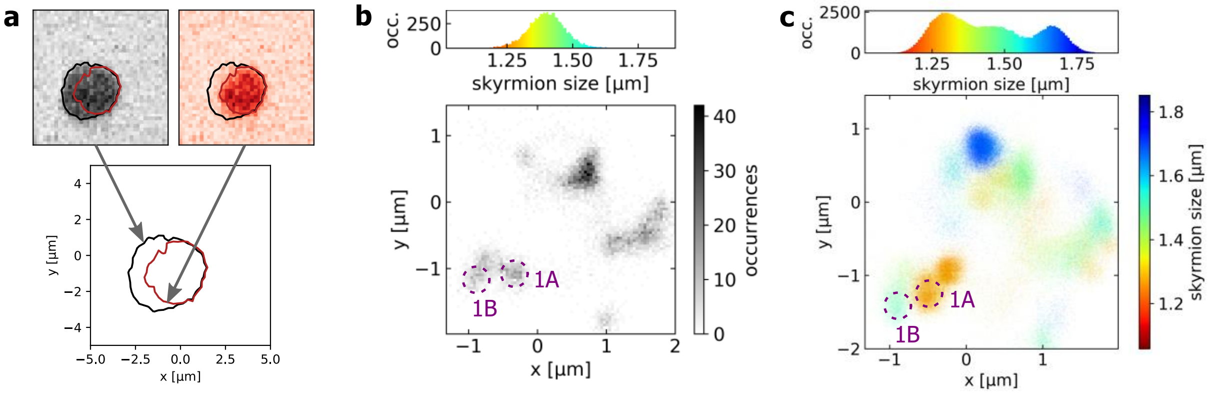

Supplementary Fig. 2. Pinning of differently sized skyrmions at same pinning site. *(a) The bottom coordinate frame shows the contours of the two distinct skyrmion sizes occurring at the same pinning site during the measurement at -37 µT. The above panels show the Kerr intensities of the respective skyrmions analogously to Fig. 4. (b) Skyrmion center occurrence probabilities at -37 µT from Fig. 2c. At the pinning site which was labeled as 1 in the main text, the skyrmion center position can vary slightly depending on the skyrmion size. Two possible positions labeled as 1A and 1B are picked which correspond to the red and black boundaries and intensities shown in (a), respectively. (c) Similarly, the two positions presented in (b) are indicated also in the size dependence plot from Fig. 2a.*

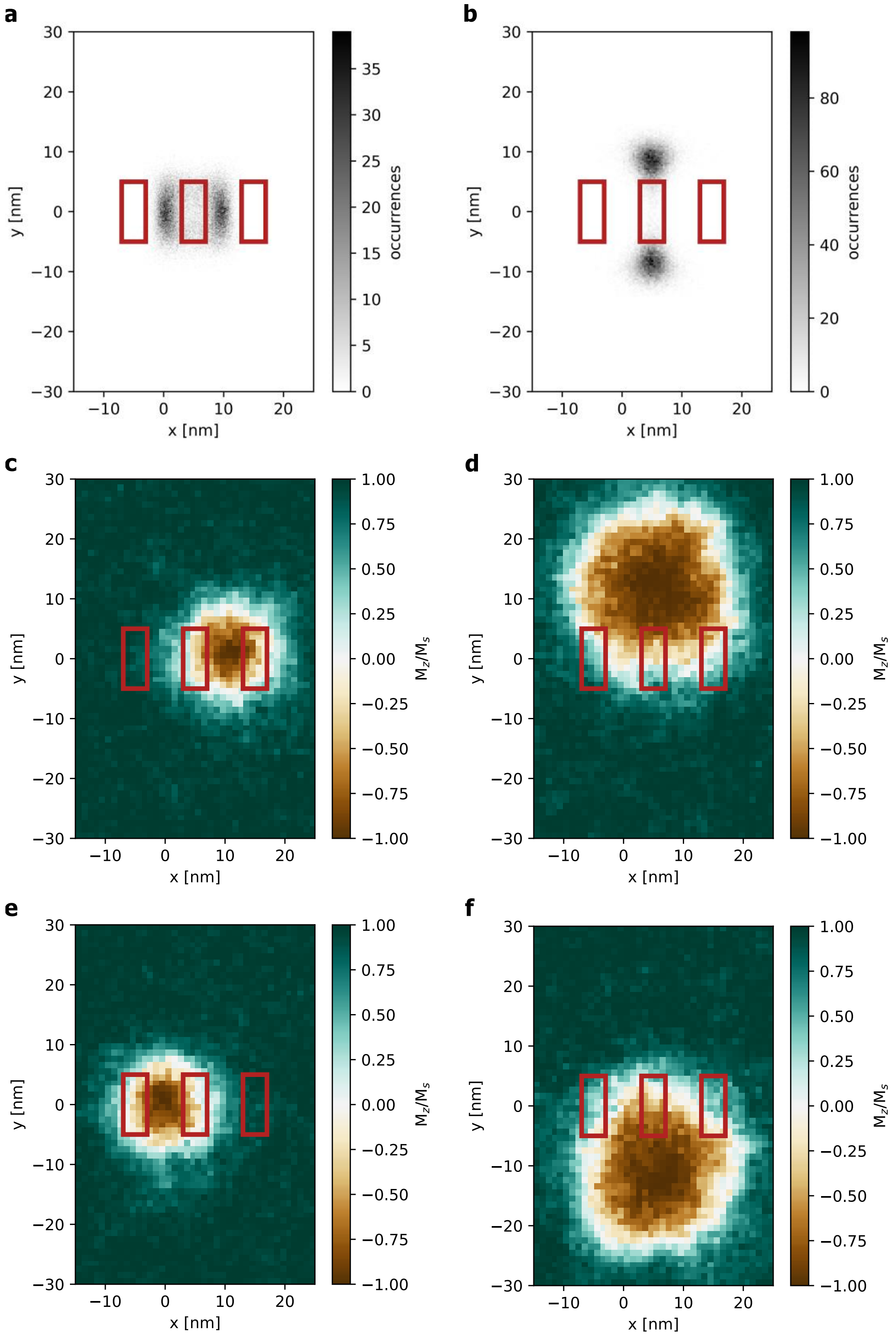

Supplementary Fig. 3. Arrangements of skyrmions in boundary pinning simulation. *(a-b) Histograms of occurring skyrmion center positions for external out-of-plane fields of (a) 0.15 T and (b) 0.05 T based on micromagnetic simulations as presented in Fig. 5. (c-f) The z-component of the unitary magnetization direction M_z/M_s of simulated skyrmions. The small skyrmions at 0.15 T in (c,e) fit between the pinning areas which are thereby covered by the SB, but not the core. In contrast, the large skyrmions at 0.05 T in (d,f) move to the top and bottom to maximize the overlap of the SB (corresponding to white region) with the area of reduced anisotropy while not covering it with their cores.*
